# Supplementary material for: Trypanosoma cruzi Sirtuin 2 as a Relevant Druggable Target: New Inhibitors Developed by Computer-Aided Drug Design
Source: Pharmaceuticals (Basel). 2023 Mar 10;16(3):428. doi: 10.3390/ph16030428 (PMC10057528; doi:10.3390/ph16030428)
Supplement: Supplementary file 1 [file pharmaceuticals-16-00428-s001.zip › pharmaceuticals-2094601-supplementary.pdf]

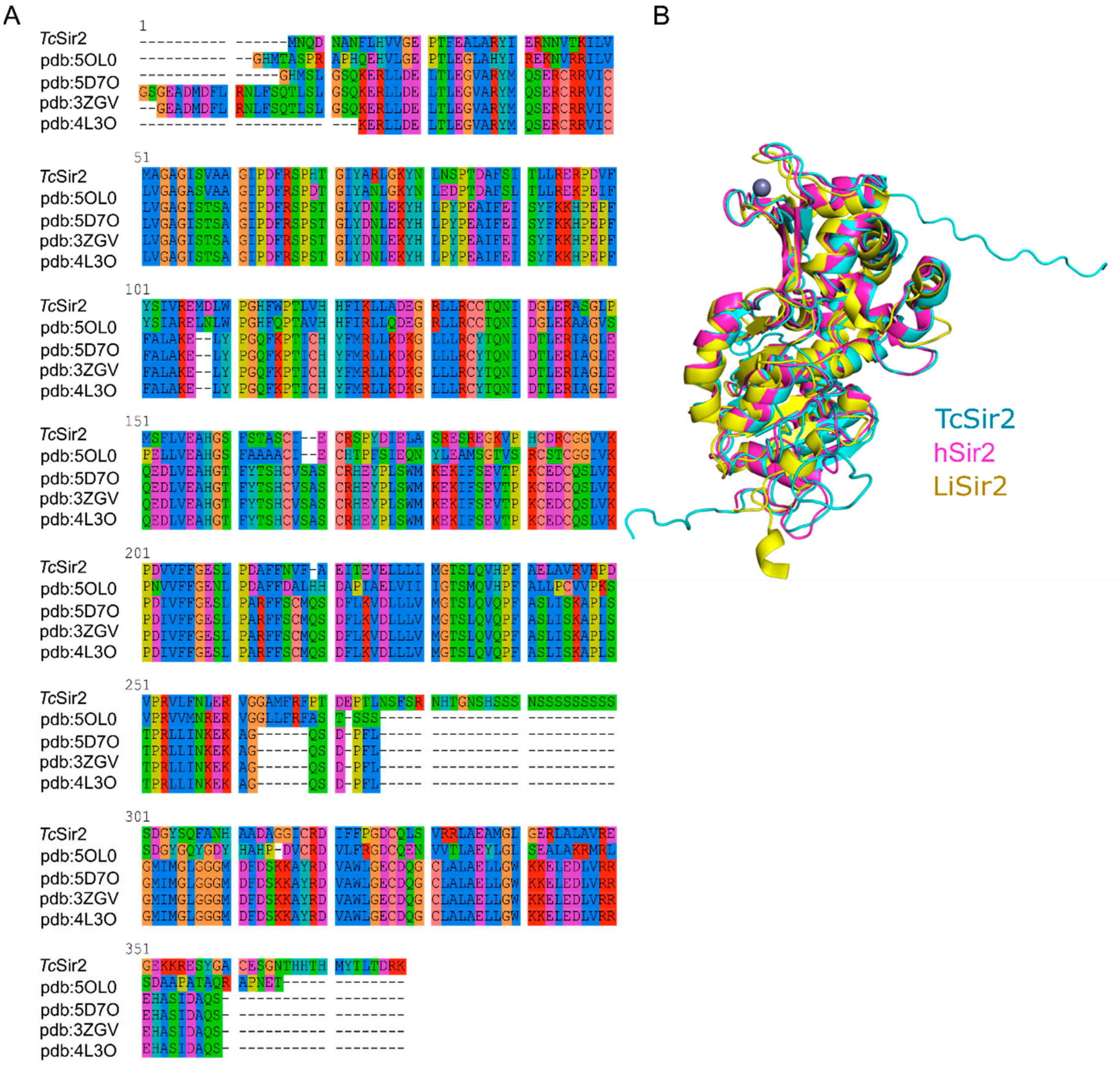

Figure S1. Comparison between trypanosomatids' Sirtuin 2 and humans. (**A**) 1D alignment between trypanosomatids' Sirtuin 2 and humans and (**B**) 3D alignment between trypanosomatids' Sirtuin 2 and humans. *Tc*Sir2 in cyan (*Trypanosoma cruzi*), *Li*Sir2 in yellow (*Leishmania infantum*; PDB: 5OL0) and hSir2 in magenta (Human; PDB: 3ZGV).

Table S1. Selection of the Sirtuin 2 crystal structures.

Organism	Similarity with TcSir2 %	PDB code	Crystal resolution	R-value free	R-value work	Ligands	NADH
**Leishmania infantum**	58.075	5OL0	1.99	0.22	0.16	Peptide linking	No
**Homo sapiens**	46.186	5D7O	1.63	0.19	0.17	No	Yes
**Homo sapiens**	46.186	4L3O	2.52	0.26	0.21	macrocyclic peptide	No
**Homo sapiens**	46.186	4X3O	1.5	0.15	0.12	myristoyl peptide	No
**Homo sapiens**	46.186	1J8F	1.7	0.26	0.23	No	No
**Homo sapiens**	46.186	6QCN	2.23	0.23	0.20	quercetin	Yes
**Homo sapiens**	46.186	5YQL	1.6	0.18	0.15	A2I (selective)	No
**Homo sapiens**	46.186	3ZGV	2.27	0.18	0.14	No	Yes
**Homo sapiens**	46.186	5MAR	1.89	0.19	0.17	1,2,4-Oxadiazole	Yes
**Homo sapiens**	46.186	4RMG	1.88	0.24	0.20	SirReal2	Yes
**Homo sapiens**	45.868	4Y6L	1.6	0.23	0.20	H3K9myr	No
**Homo sapiens**	45.798	4R8M	2.1	0.27	0.22	BHJH-TM1	No
**Homo sapiens**	45.763	3ZGO	1.63	0.19	0.16	No	No

PDB: Protein Bank Data (https://www.rcsb.org/).

Table S2 Interaction regions of different Sir2 organisms. *Tc*Sir2 (*Trypanosoma cruzi*); *Li*Sir2 (*Leishmania infantum*); hSir2 (*Homo sapiens*) and *Tb*Sir2 (*Trypanosoma brucei*).

**Organism**	**Catalytic site**			**Organism**	**Catalytic site**		
	**C**	**B**	**A**		**C**	**B**	**A**
***Tc*Sir2 (model)**	Phe49	Gly37	Val42	***Li*Sir2 (PDB:5OL0)**	Phe51	Gly39	Cys235
	Arg50	Ala38	Ala43		Arg52	Ala40	Gly333
	Ile56	Gly39	Phe237		Ile58	Gly44	Ser334
	Pro68	Ile40	Asn238		Ala73	Ile48	Gln336
	Phe72	Met212	Leu239		Phe74	Asp50	Glu337
	Val88	Gly213	Glu240		Ile89	Gly216	Asn338
	Asp125	Thr214	Cys308		Leu95	Thr217	
	Leu93	Ser215	Glu309		Gln124	Ser218	
	Asn123	Glu217			Asn125	Asn241	
	Ser215	Val242			Ile126	Arg242	
	Gln217	Gln287			His144	Glu243	
	Val218				Val221		
**hSir2 (PDB:3ZGV)**	Phe96	Gly84	Lys187	***Tb*Sir2 (model)**	Phe49	Gly37	Ala43
	Leu103	Ala85	Asn286		Arg50	Ala38	Phe307
	Tyr104	Gly86	Glu288		Leu56	Gly39	Pro308

Ile118	Thr89	Trp30	Tyr57	Gly213	Gly909
Phe119	Ile93	Leu321	Phe72	Thr214	Asp310
Leu134	Pro94	Gly322	Ile87	Ser215	Cys311
Asn160	Asp95	Glu323	Met89	Asn238	
Gln167	Arg97	Leu324	His142	Leu239	
Met168	Ser98			Glu240	
His187	Gly261			Val242	
Val266	Thr262				
	Ser263				
	Leu264				
	Pro264				

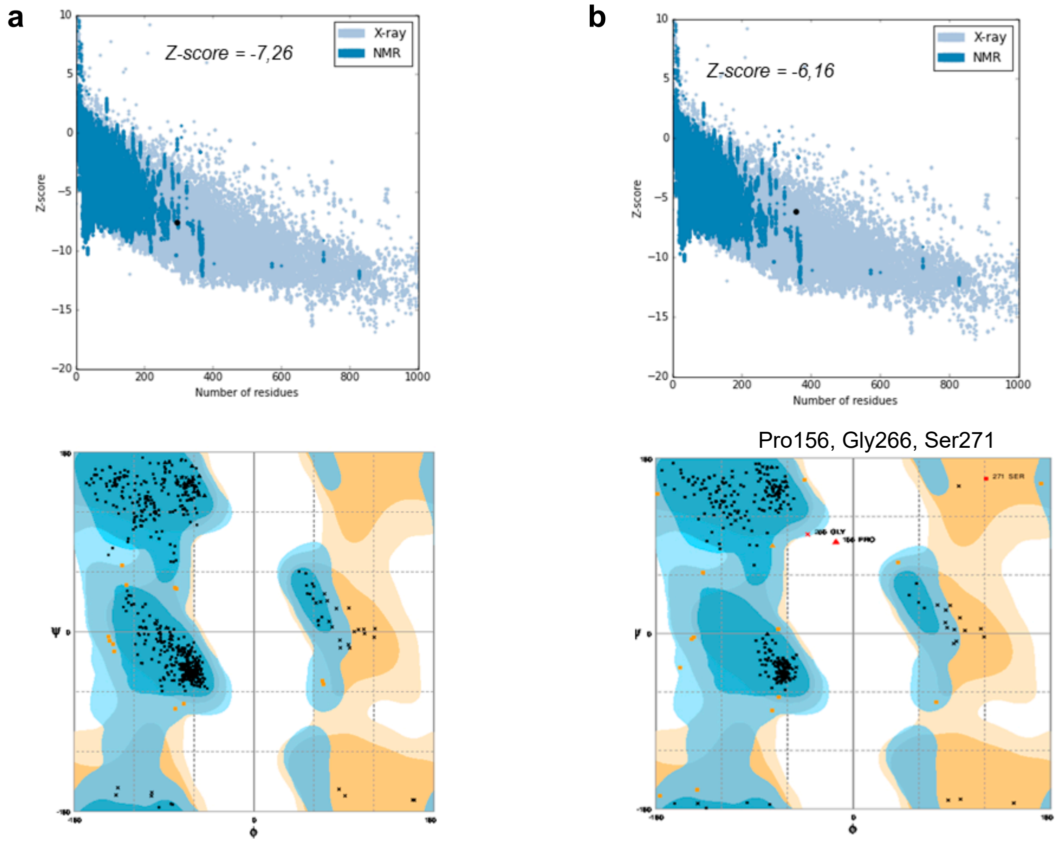

Figure S2. Ramachandran and Z-score validation*. (**a**) *Tc*Sir2 before MD (0.5% outlier and Z-score -7.26) and (**b**) *Tc*Sir2 after MD (0.0% outlier and Z-score -6.16). * These give an assessment of the overall quality of the structure as compared with well refined structures of the same resolution and also highlight regions that may need further investigation. The PROCHECK programs are useful for assessing the quality not only of protein structures in the process of being solved but also of existing structures and of those being modelled on known structures.

Supplementary methods – Molecular dynamics simulation parameters

Initially, the relaxation of the system was performed using Steepest Descent and the limited-memory Broyden-Fletcher-Goldfarb-Shanno algorithms in a hybrid manner. The simulation was performed under NPT ensemble for 5 ns implementing the Berendsen thermostat and barostat methods. A constant temperature of 310 K was maintained throughout the simulation using the Nose-Hoover thermostat algorithm and Martyna-Tobias-Klein Barostat algorithm to maintain 1 atm of pressure, respectively. After minimization and relaxation of the system, we proceeded with a single production step of 1 μs. The representative structure was selected by clustering the structures from the RSMD values, using 1 Å as a cut-off. Interactions and distances were determined using the Simulation Event Analysis pipeline implemented in Maestro (Maestro 2019v2). The employed geometric criteria for protein-ligand H-bond is the distance of 2.5 A° between the donor and acceptor atoms (D — H···A); a donor angle of ≥120° between the donor-hydrogen-acceptor atoms (D — H···A); and an acceptor angle of ≥90° between the hydrogen-acceptor-bonded atom atoms (H···A — X). Similarly, protein-water or water-ligand H-bond are: a distance of 2.8 A°between the donor and acceptor atoms (D—H···A); a donor angle of ≥110° between the donor-hydrogen-acceptor atoms (D—H···A); and an acceptor angle of ≥90° between the hydrogen-acceptor-bonded atom atoms (H···A—X). Non-specific hydrophobic interactions are defined by hydrophobic sidechain within 3.6 A° of the ligand's aromatic or aliphatic carbons and π-π interactions required two aromatic groups stacked face-to-face or face-to-edge, within 4.5 A° of distance.

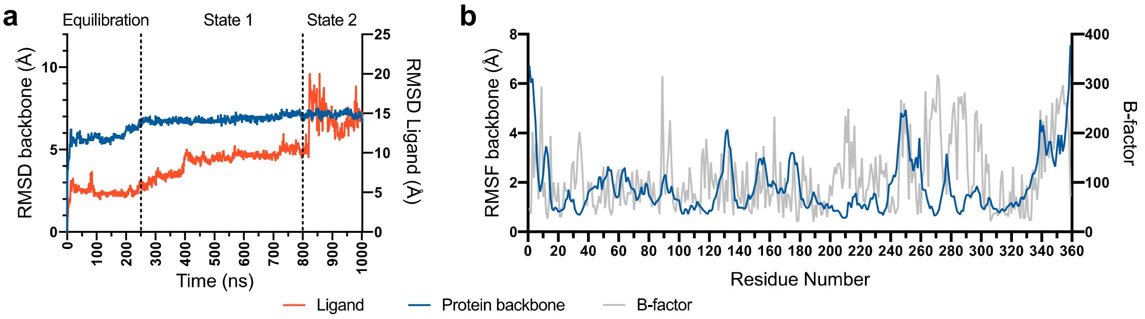

Figure S3. (**a**) *Tc*Sir2 and NAD^+ RMSD values along MD trajectory, showing two stages the protein explored along the simulation time, after equilibration; (**b**) Root mean squared fluctuation by residues (RMSF) compared against the experimental B-factor.

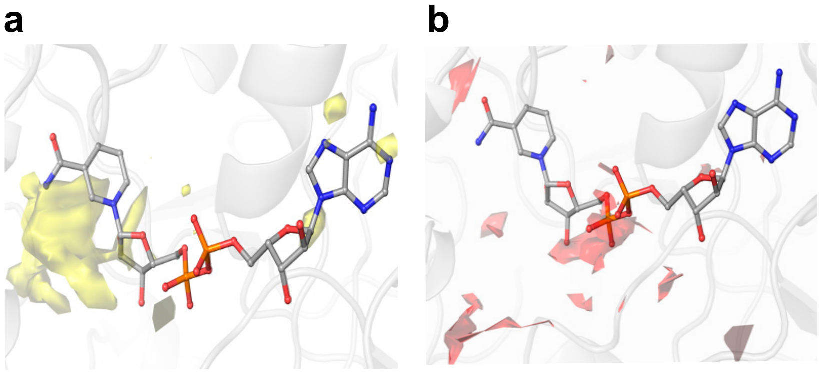

Figure S4. Molecular Interaction Field analysis. (**a**) Dry probe for hydrophobic regions in yellow and (**b**) O_2 probe for acceptor and acceptor regions in red.

Supplementary methods – Enzyme kinetic characterisation

The enzyme kinetic assay for *Tc*Sir2, based on a previous publication[1] was divided into three steps. In the first one, the recombinant *Tc*Sir2, NAD^+ and the peptide substrate (Abz-Gly-Pro-AcetylLys-Ser-Gln-EDDnp) were incubated for 15 min at 37° C. In the second step, in order to stop the reaction, 12 mM nicotinamide was added to the reaction medium. Finally in the third step trypsin was added and the reaction medium incubated for 30 min at 37° C, as previously described[1]. Trypsin cleaves this substrate in the linkage between lysine and serine only when the acetyl moiety had been previously removed by *Tc*Sir2. As a control, it was observed that trypsin did not cleave the substrate when lysine was acetylated[2]. The Michaelis-Menten constant (K_m) for the NAD^+ (25 μM, $r^2 = 0.99$, Fig. S5a) and the peptide substrate (2.1 μM, $r^2 = 0.97$, Fig. S5b) were determined using seven different substrate concentrations, whereas one of them was kept constant.

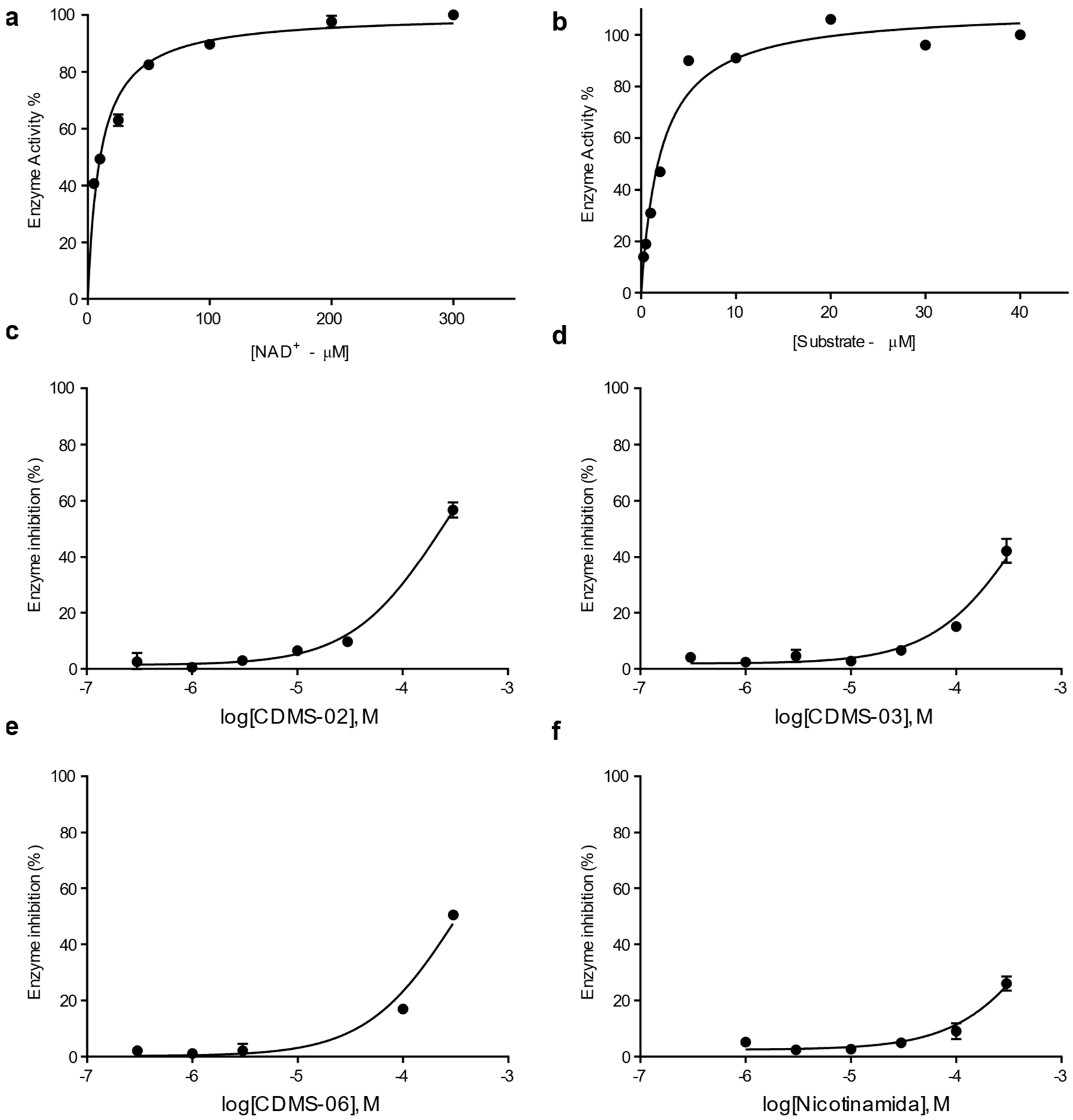

Figure S5. K_m calculation for formation ADP-ribose (**a**) and the deacetylation the peptide (**b**) followed by CDMS-02 (**c**), CDMS-03 (**d**), CDMS-06 (**e**) and Nicotinamide (**f**) dose-response curves for enzymatic inhibition on *Tc*Sir2.

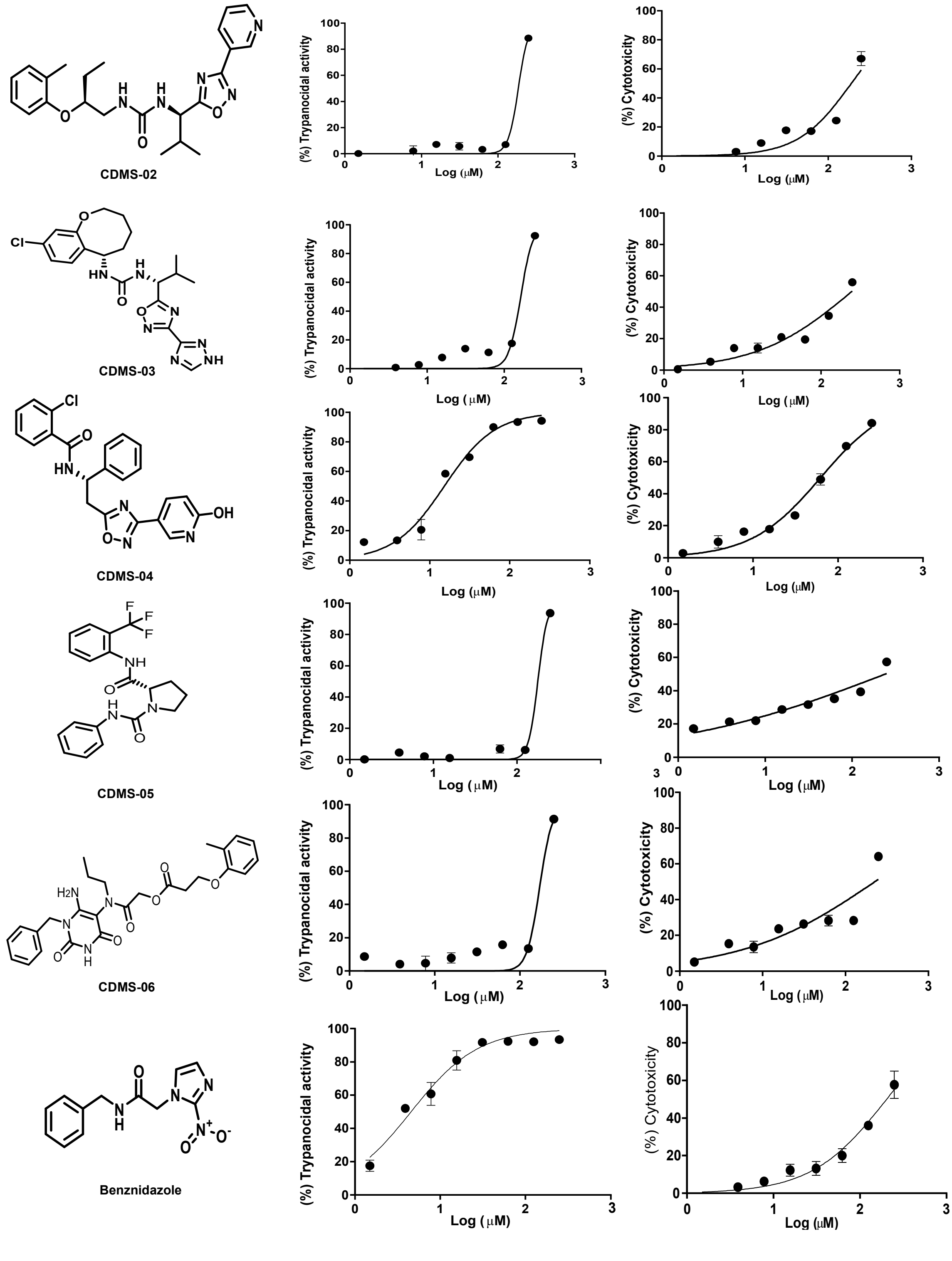

Figure S6. Chemical structures for each tested compound CDMS-02 to CDMS-06, followed by dose-response curves for trypanocidal activity on amastigote stage and cytotoxicity on mammalian cells.

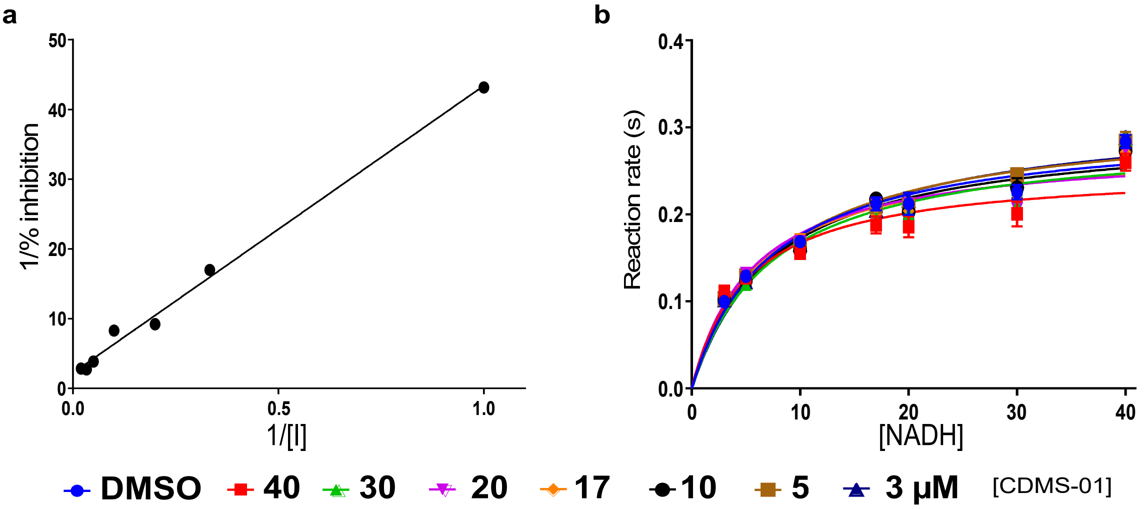

Figure S7. CDMS-01 mechanism inhibition. (**a**) Plot for determination of the Ki (Ki = 21.5 µM and r^2 = 0.989); (**b**) mechanism inhibition of CDMS-01. Reaction rate, in seconds, and substrate concentration were analysed in the presence of different concentrations of CDMS-01.

**Calculated by reaction speed**								
Compound (µM)	**DMSO**	**40**	**30**	**20**	**17**	**10**	**5**	**3**
V_{max} (µM)	0.29	0.29	0.29	0.29	0.29	0.29	0.29	0.29
K_m (µM)	6.60	8.84	7.49	6.74	6.92	6.98	6.41	6.58

Table S3. F-test[2] results support a competitive inhibition mechanism as observed by the differences in the fluorescent unit.

References

1. Moretti, N. S. *et al.* Characterization of Trypanosoma cruzi Sirtuins as Possible Drug Targets for Chagas Disease. *Antimicrob Agents Chemother* **59**, 4669–4679 (2015).
2. Copeland, R. A. *Evaluation of Enzyme Inhibitors in Drug Discovery*. (John Wiley & Sons, Inc., 2013). doi:10.1002/9781118540398.
